# Rapid stability of ferroelectric polarization in the Ca, Ce hybrid doped BaTiO_3_ ceramics

**DOI:** 10.1038/srep38354

**Published:** 2016-12-22

**Authors:** Shujuan Liu, Lixue Zhang, Jiping Wang, Xiujing Shi, Yingying Zhao, Dawei Zhang

**Affiliations:** 1 State key Laboratory for Mechanical Behavior of Materials, School of Materials Science and Engineering, Xi’an Jiaotong University, Xi’an 710049, China

## Abstract

In this work, we report a rapid stability phenomenon of ferroelectric polarization in the Ca, Ce hybrid doped BaTiO_3_ ceramics (BCaxT+BTCe8) (x = 10, 20, 24, 30 mol%) prepared by separate doping Ca^2+^ and Ce^4+^ ions. Double hysteresis loops are identified in the aged BCaxT+BTCe8 samples; meanwhile, the polarization of these loops present a rapid decrease within very short aging time (about 1 h), and then the polarization remains almost unchanged over the followed ~1000 h. This phenomenon is not reported in previous researches. Raman scattering spectrum indicates that oxygen vacancies are generated because of Ca^2+^ ions entering into Ti sites partly in the BCaxT+BTCe8 samples, and then the oxygen vacancies are quantitatively characterized by half of the Ce^3+^ content through the XPS test. The emergence of the aging phenomenon is explained through the 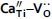 defect dipole reorientation mechanism. The larger radius of Ca^2+^ ions is further discussed as a possible reason for the rapid stability phenomenon of ferroelectric polarization. It may provide an effective design method from the viewpoint of the ionic radius to accelerate polarization stability, and thus to facilitate the possible practical applications of the aging effect.

Ferroelectric materials are widely used as capacitors, transducers, sensors, actuators and refrigerant because of their extensive function effects such as dielectric response, piezoelectric, electrostrictive and electrocaloric effects^1^,^2^,^3^,^4^. However, for the application of ferroelectric materials, most of these physical properties are subjected to changes with time (namely aging effect)^5^. On one hand, the aging effect is one of the most ineluctable obstacles to the reliability and stability of ferroelectric materials, because the properties like polarization reduces over time^5^,^6^,^7^. On the other hand, some studies on the aging behaviour have also found that the utilization of the aging effect can lead to a very large recoverable electro-strain^8^,^9^,^10^,^11^. Usually, such kind of aging-associated property requires an aging treatment. In either case, a long aging time (from hundreds of to thousands of hours) is necessary to ensure a stable performance^9^,^12^,^13^,^14^. Thus there need much work to predict the lifespan of the physical properties.

Many researchers dedicate to study the lead zirconate titanate (PZT) family^12^,^15^, the barium titanate (BT) family^6^,^8^,^9^,^13^,^14^,^16^,^17^,^18^,^19^,^20^,^21^,^22^ and some other lead-free compositions^23^ to develop reasonable theories to explain the aging phenomenon. There are three theories that have been proposed: grain boundary effect, domain wall effect and volume effect^5^,^8^. From these aging theories, the “strength” of aging effect can be controlled by choosing type of the doping ions and the base system^13^,^21^. However, the effective ways or compositions to reduce aging time, and mostly importantly, to fast reach the stability time of the properties remain awaiting.

Ca and Ce are those chemical dopants which can be incorporated at the Ba-sites or at the Ti-sites in BaTiO_3_ when sintered in air. Some researchers have investigated the aging behavior of the Ca doped, Ca, Mn hybrid doped or Ca, Zr hybrid doped BaTiO_3_ ceramics^21^,^22^,^24^,^25^. Yun *et al*. use the Raman spectrum at a high frequency peak about ~827 cm^−1^ to show that, when the Ca^2+^ ions enter into Ti sites in BaTiO_3_ and form 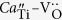 defect dipoles, the aging phenomenon with double hysteresis loops will appear^24^. Similar Raman results are evidenced by Puli *et al*. when Ca^2+^ ions enter into Ti sites in Bi-BCT system^25^. While, for Ce doped BaTiO_3_ ceramics, the researchers usually focus on their piezoelectric properties^26^. Lu *et al*. show Raman evidence at the ~840 cm^−1^ peak for the Ba-Site Ce^3+^ in BaTiO_3_^27^. Few study are about the Ca, Ce hybrid doped BaTiO_3_ ceramics and their aging behavior.

In this paper, we report a rapid ferroelectric polarization stability phenomenon (only aged about 1 h) in the Ca, Ce hybrid doped BaTiO_3_ ceramics (BCaxT+BTCe8). The rapid stability about hysteresis loops is discussed from a defect dipole reorientation mechanism and the ionic radius of the dopants. Our results may provide a possibility for accelerating aging treatment, and thus make the utilization of the properties associated with the aging effect more efficient, stable and securer.

## Results and Discussion

### XRD patterns results

**Figure 1:**
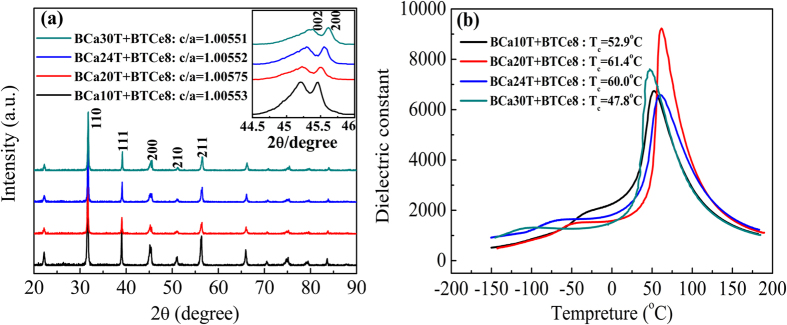
The XRD patterns (**a**), the temperature dependence of relative permittivity at a frequency of 1 kHz (**b**) of the BCaxT+BTCe8 samples.

Figure 1(a) showed the XRD patterns of the BCaxT+BTCe8 samples. Splitting peaks of the (002) and (200) appeared in all these samples. Such splitting illustrates the samples are illustrate the ferroelectric perovskite phases with the tetragonal structure at the room temperature. So all the aging-associated studies were performed at the room temperature where the samples are in tetragonal phases. The c/a ratio of the BCa20T+BTCe8 samples is 1.00575, showing the maximal c/a ratio value among all the BCaxT+BTCe8 samples.

### Phase transition point

Figure 1(b) showed the temperature dependence of the relative permittivity at a frequency of 1 kHz for the BCaxT+BTCe8 samples. As shown in Fig. 1(b), during cooling process, the first permittivity peak corresponds to the cubic–tetragonal phase transition, and the corresponding temperature is called Curie temperature (*T*_*c*_); the temperature of the second permittivity peak represents the tetragonal–orthorhombic phase transition temperature (*T*_*o*_). The *T*_*c*_ value is respectively 52.9, 61.4, 60.0 and 47.8 °C for BCa10T+BTCe8, BCa24T+BTCe8, BCa24T+BTCe8 and BCa30T+BTCe8 samples, all higher than room temperature. It is further found that the *T*_*c*_ increases firstly and then decreases with the increase of the Ca content. The maximum *T*_*c*_ value 61.4 °C appears at BCa20T+BTCe8 sample. The tetragonal–orthorhombic phase transition temperature (*T*_*o*_) strongly decreases with the Ca concentration increasing. According to the previous reports about the effect of Ca on the transition temperature^28^,^29^, most of Ca in our samples is successfully incorporated as Ca^2+^ into Ba sites in our samples, following the separate doping BCaxT samples.

### Rapid ferroelectric polarization stability phenomenon

**Figure 2:**
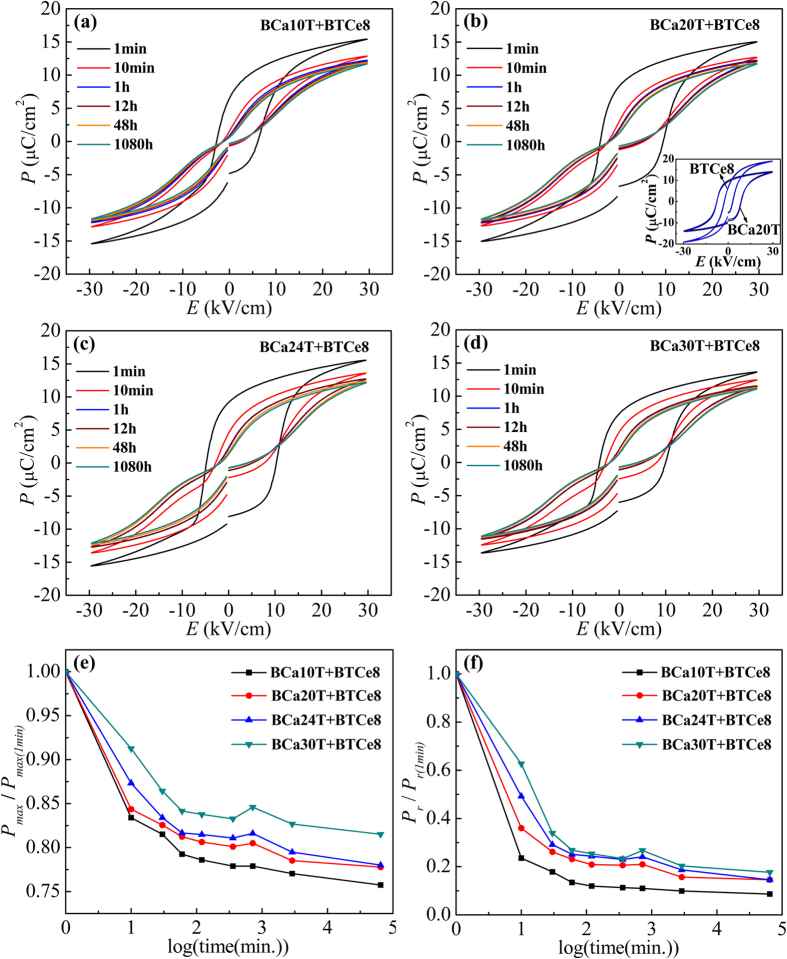
The measured hysteresis loops of the BCaxT+BTCe8 samples after aging 1 min, 10 min, 1 h, 12 h, 48 h and 1080 h (**a**–**d**), the value of *P*_*max*_/*P*_*max(1min)*_ (**e**), and the value of *P*_*r*_/*P*_*r(1min)*_ (**f**) of the BCaxT+BTCe8 samples changing with the logarithmic aging time. Inset of the figure (**b**) shows the measured hysteresis loops of the separate doping samples (represented by BCa20T and BTCe8) before and after aging 1 h at room temperature.

Ferroelectric property was shown in the Fig. 2. Figure 2(a–d) showed the measured P-E hysteresis loops of the BCaxT+BTCe8 samples before and after their aging treatment. Without aging, all the samples show the squared hysteresis loops; while obvious double loops are identified after the aging treatment. It is worth noting that all these samples with the similar P-E hysteresis loops characteristics show a rapid loop-shrinking along the P-axis within very short aging time 1 h (under E = 30 kV/cm). However, when continuously increasing aging time, the double loops remain almost unchanged. The polarizations present a same trend which have a sharp decrease and then become smooth. Thus an obvious rapid polarization stability phenomenon arises in our samples. Interestingly, Unlike the BCaxT+BTCe8 samples, the P–E loops of those separate doping samples including BCa20T and BTCe8 remain almost unchanged before and after aging treatment (as shown in inset of Fig. 2(b)). They show the squared hysteresis loops after aging 1 h, without the obvious aging phenomenon as the hybrid doped samples. This indicates that the hybrid doped BCaxT+BTCe8 samples may have some differences in the substitution sites from the separate doping samples BCaxT and BTCe8.

*P*_*max*_/*P*_*max(1min)*_ and *P*_*r*_/*P*_*r(1min)*_ were used to characterize the rapid ferroelectric polarization aging stability phenomenon more clearly, as shown in Fig. 2(e,f). In the Fig. 2(e), the degree of the switched domains when applied electric field at different logarithmic aging time was represented by *P*_*max*_/*P*_*max(1min)*_. It can be seen that, a large and rapid decrease of the *P*_*max*_/*P*_*max(1min)*_ in a short aging time (about 1 h, shown by the fourth point in the figure) appears at a rate of 15.8~26.0% per hour. And then, with the increase of the aging time, the *P*_*max*_/*P*_*max(1min)*_ remains almost unchanged at a decreasing rate of 0.002~0.005% per hour for all the BCaxT+BTCe8 samples. This means the domain stabilization time is reduced to 1 hour in our study when applied electric field. Besides, with the increase of the Ca concentration, the decrement of *P*_*max*_/*P*_*max(1min)*_ becomes smaller, which shows that the switched domain reduces when doping more Ca. In the Fig. 2(f), the degree of the switched domains when removed electric field at the different logarithmic aging time was represented by *P*_*r*_/*P*_*r(1min)*_. One can see the same laws of *P*_*r*_/*P*_*r(1min)*_ as the *P*_*max*_/*P*_*max(1min)*_. That is to say, the samples present a rapid domain stabilization phenomenon (also about 1 h) when removed electric field.

For comparison, some previous works about the stability time of the dielectric properties (under small field signal) and the ferroelectric polarization (under large field signal) are summarized in Table 1^ ^9,12,13,14,23,30,31,32,33,34,35,36. As shown in Table 1, for the dielectric properties, the aging stability time of the Fe, La doped BT, and the Fe doped PZT is respectively above 100 min (~1.7 h) and 1000 min (~16.7 h). Compared with this, for the ferroelectric polarization, the aging stability usually goes through a long time in previous reports. For example, for the Mn doped BST, Mn doped BT, Al doped BT, Cu doped KNN, and Fe doped PZT ceramics, the aging stability time is all above hundreds to thousands of hours. Also, there are some researchers have reported that in the Cu, Fe doped PT ceramics, the aging stability time is about hours to days according to the computational simulation results. All in all, the stability time 1 h of the ferroelectric polarization in our work has not been reported before.

### Raman spectra and XPS results

**Figure 3:**
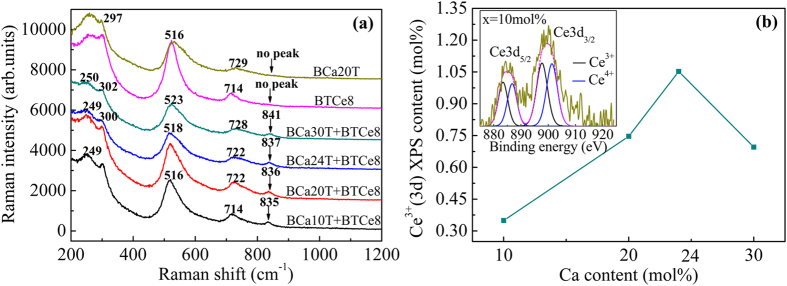
Raman spectra of the BCaxT+BTCe8 samples and the separate doping samples (represented by BCa20T and BTCe8) after aging 1 h (**a**), the Ce^3+^ (3d) XPS content with the Ca content increasing (**b**). The inset in (**b**) shows the Ce3d XPS spectra and its Lorentzian dividing results for the BCa10T+BTCe8 samples.

The BCaxT+BTCe8 samples were investigated by Raman spectroscopy to show the relationship between the lattice dynamics and the doping positions in this study. As shown in Fig. 3(a), there are four Raman bands in all the samples being similar to the reported single-crystal and ceramic BaTiO_3_^37^: A1(TO_2_), B1+E(TO+LO), A1(TO_3_), and A1(LO_3_)+E(LO_3_) with peaking at ~249, ~300, ~516, and ~722 cm^−1^, respectively. Obviously, the higher frequency Raman band at ~840 cm^−1^ appears in the spectra of all the BCaxT+BTCe8 samples, but does not appear in the separate doping samples (represented by BCa20T and BTCe8). This band at 830~840 cm^−1^ was found in many inequitable doping BaTiO_3_ systems such as La^3+^ ions, Nd^3+^ ions, Eu^3+^ ions, Ce^3+^ ions at Ba sites, and Ca^2+^ ions at Ti sites in the previous reports^24^,^25^,^27^,^38^,^39^,^40^. According to previous works and the ion size factor in BaTiO_3_ system, this mode at about ~840 cm^−1^ is attributed to an internal deformation of the BO_6_ octahedron caused by the charge difference of different types of ions at equivalent sites in BaTiO_3_^17^,^24^,^25^,^27^,^38^,^39^,^40^,^41^. Correspondingly, the weak peak at ~840 cm^−1^ in BaTiO_3_ doping with Ce may be attributed to Ce entering Ba sites as Ce^3+^, while it has no connection with Ce^4+^ at Ti sites^27^. The weak peak in BaTiO_3_ doping with Ca may be attributed to partial migration of Ca^2+^ from Ba to Ti sites^24^,^25^. So in our study, the Raman test results indicate that Ca is successfully incorporated as Ca^2+^ into Ba sites, and that Ce is successfully incorporated as Ce^4+^ into Ti sites in the separate doping samples, while the Ca, Ce hybrid doped samples have different substitution sites. That is to say, Ce enters into Ba sites partly as Ce^3+^ or Ca enters into Ti sites partly as Ca^2+^ in the BCaxT+BTCe8 samples. Because of the multivalence of Ce^3+^/Ce^4+^ at Ba/Ti sites showing different binding energy in the XPS spectra^42^,^43^, we can distinguish the Ce^3+^ peak and the Ce^4+^ peak from the Ce3d XPS spectra. Meanwhile, the area ratio of these two peaks can also be obtained. Then from the XPS quantification report, we can know the total molar fraction of the Ce3d. Thus the exact Ce^3+^ content in the BCaxT+BTCe8 samples can be got through the area ratio of the Ce^3+^ and Ce^4+^ peak and the total molar fraction of the Ce3d. As show in Fig. 3(b), with the Ca content increasing, the Ce^3+^(3d) XPS content is 0.17, 0.37, 1.05, and 0.70 mol% respectively for the BCaxT+BTCe8 samples.

### Explanation of the emergence of the aging phenomenon based on the charge balance and chemical composition balance

**Figure 4:**
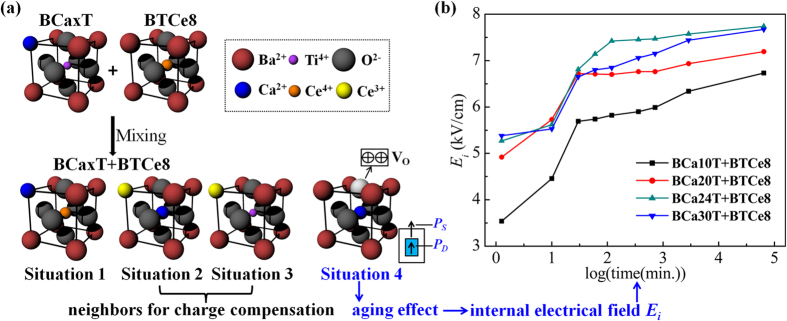
The four possible situations (**a**) (Situation1: one Ca^2+^ ion is substituted for A-site Ba^2+^ ions, meanwhile one Ce^4+^ ion is substituted for B-site Ti^4+^ ions; Situation 2: one Ce^3+^ ion is substituted for A-site Ba^2+^ ions, meanwhile one Ca^2+^ ion is substituted for B-site Ti^4+^ ions; Situation 3: one Ce^3+^ ion is substituted for A-site Ba^2+^ ions; Situation 4: one Ca^2+^ ion is substituted for B-site Ti^4+^ ions causing the formation of one oxygen vacancy) and the internal electrical field *E*_*i*_ (**b**) of the BCaxT+BTCe8 samples after aging 1 min, 10 min, 30 min, 1 h, 2 h, 6 h, 12 h, 48 h and 1080 h.

Considering the above research results (obvious double hysteresis loops after aging and the weak 840 cm^−1^ Raman band), we will propose here four possible situations in the BCaxT+BTCe8 samples as shown in Fig. 4(a), which can explain the emergence of the aging phenomenon in our samples. Firstly, one Ca^2+^ ion is substituted for A-site Ba^2+^ ions, meanwhile one Ce^4+^ ion is substituted for B-site Ti^4+^ ions. According to the previous reports about Ba(Ti,Ce)O_3_^26^, the Ce^4+^ at Ti sites can help to stabilize the Curie temperature (*T*_*c*_) (as evidenced in Fig. 1(b)). If all the Ce is incorporated at the Ba sites as Ce^3+^, the *T*_*c*_ would drop. And when the *T*_*c*_ reduces to below the room temperature, the ferroelectric aging effect will disappear due to the sample presenting a paraelectric phase rather than a ferroelectric phase at room temperature. Secondly, one Ce^3+^ ion is substituted for A-site Ba^2+^ ions, meanwhile one Ca^2+^ ion is substituted for B-site Ti^4+^ ions. Thus a positive charge is lack to meet the charge balance. Thirdly, one Ce^3+^ ion is substituted for A-site Ba^2+^ ions, so that a positive charge is unnecessary for the charge imbalance. The second and the third situation should be neighbors for charge compensation. Fourthly, from the chemical composition balance, there remains a Ca^2+^ ion. The remaining Ca^2+^ ion is substitute for B-site Ti^4+^, causing the formation of one oxygen vacancy to balance the charge misfit. Thus the fourth situation about the formation of oxygen vacancy is considered to induce the obvious aging effect in our study.

Besides, the quantitative information of oxygen vacancies can be obtained from above proposed situations. Considering the second, the third and the forth situation, if there are two Ce^3+^ ions are substituted for A-site Ba^2+^ ions, there must be two Ca^2+^ ions are substituted for B-site Ti^4+^ ions. One of the Ca^2+^ ions is for charge balance; another remaining Ca^2+^ ion which causes the formation of one oxygen vacancy is for the chemical composition balance. Therefore, the oxygen vacancy generated by the Ca^2+^ ions substitution at the B-site can be quantitatively characterized by half of the Ce^3+^ content. So the oxygen vacancy content generated by the Ca^2+^ ions substitution at the B-site is 0.085, 0.185, 0.525, 0.35 mol% respectively with the Ca content increasing from the Ce^3+^ (3d) XPS content in the Fig. 3(b). Thus we can quantitatively characterize the content of oxygen vacancies through charge balance, chemical composition balance, and half of the Ce^3+^ content by XPS test.

The double hysteresis loops related to the aging phenomenon could be well explained by the defect dipole reorientation mechanism, followed by the recent symmetry-conforming short-range ordering (SC-SRO) principle^8^,^44^,^45^. As the fourth situation mentioned, 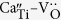 defect dipoles form in the unit cell. After aging, the 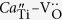 defect dipoles align along the spontaneous polarization (*P*_*S*_) direction because of the oxygen vacancy migration; and the aligned 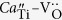 defect dipoles form defect dipole polarization (*P*_*D*_)^8^,^16^,^17^. According to the SC-SRO principle, the 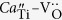 defect dipoles and the associated *P*_*D*_ cause reversible domain switching, and the double hysteresis loops associated with aging are observed under the periodic electric-field drive. Correspondingly, during the P−E loop measurement, the un-switched defect symmetry and associated defect dipoles generate an internal electrical field *E*_*i*_ for stabilizing the domain pattern. The *E*_*i*_ is calculated by the equation of *E*_*i*_ = (*E*_*1*_ + *E*_*2*_)/2, where *E*_*1*_ and *E*_*2*_ are the peak field of forward and backward domain switching processes^46^. In our study, *E*_*i*_ was calculated respectively by the loops. As shown in Fig. 4(b), the defect dipoles field *E*_*i*_ build up in all BCaxT+BTCe8 samples. All the *E*_*i*_ present a large and rapid increase over the logarithmic function of aging time in the initial period (about 1 h, shown by the fourth point in the figure), and then saturates to a stable value. This result about *E*_*i*_ and *P*_*D*_ is consistent with the laws of *P*_*max*_ and *P*_*r*_ in sense of the rapid aging stability.

### Origin of the rapid ferroelectric polarization stability phenomenon

Then the causes of the rapid ferroelectric polarization stability phenomenon should be discussed. Due to the size of the oxygen octahedral gap having great influence on the movement of oxygen vacancy, the ionic radius is considered to be an important factor on aging stability phenomenon in this study. Here we compare the aging effect of our samples with other acceptor-doped ions in BaTiO_3_ systems. We easily find that the aging stability time of polarization (about 1 h) of the BCaxT+BTCe8 samples in our study is much shorter than those reported value in doping with Mn samples (hundreds of hours)^13^,^14^. Here we propose a possible reason for this phenomenon. Although the similar c/a ratios between our samples and the Mn doped samples indicate the similar thermodynamic driving force for the migration of oxygen vacancy^13^, the kinetic migration condition of oxygen vacancy in the oxygen octahedral of these systems is different due to the ionic size difference between Ca and Mn. The similar thermodynamic driving force leads to the initial aging effect in these two systems, and then the driving force reduces due to the redistribution of oxygen vacancy after the initial aging. With the aging time further increases, the reduced force is not large enough for more oxygen migration as to overcoming the barrier from the Ca^2+^ ions, as the radius of Ca^2+^ ions (R_Ca_^2+^=1 Å) is larger than the Mn^3+^ ions (R_Mn_^3+^=0.645 Å). Therefore, the migration is stopped soon and the aging reaches stabilization soon in our hybrid doped samples. Thus the larger ionic radius is a possible reason for the rapid ferroelectric polarization stability phenomenon. More experimental data and computational simulation can be used to verify these possible causes.

## Conclusion

In summary, the ferroelectric polarization aging behavior of the Ca, Ce hybrid doped samples (BCaxT+BTCe8) were investigated in this study by the measurement of the hysteresis loops (under E = 30 kV/cm) and corresponding characterization parameters *P*_*max*_/*P*_*max(1min)*_, *P*_*r*_/*P*_*r(1min)*_ and the internal electrical field *E*_*i*_ changing over the logarithmic function of aging time. Obvious double loops were identified in the aged BCaxT+BTCe8 samples. Meanwhile, these hybrid doped samples show a rapid ferroelectric polarization stability phenomenon only after aging about 1 h, which is not reported in previous researches. Raman scattering spectrum indicates that the Ce enters into Ba sites partly as Ce^3+^ or Ca enters into Ti sites partly as Ca^2+^ in the BCaxT+BTCe8 samples. And through the XPS test, the exact Ce^3+^ content in the BCaxT+BTCe8 samples is obtained. Based on the charge balance and chemical composition balance, four possible situations in the BCaxT+BTCe8 samples are proposed: (1) one Ca^2+^ at Ba sites, meanwhile one Ce^4+^ at Ti sites; (2) one Ce^3+^ at Ba sites, meanwhile one Ca^2+^ at Ti sites; (3) one Ce^3+^ ion at Ba sites; (4) one Ca^2+^ ion at Ti sites. The fourth situation about the formation of oxygen vacancy because of the Ca^2+^ ions partly entering into Ti^4+^ sites induces obvious aging effect. The oxygen vacancies are quantitatively characterized by half of the Ce^3+^ content through XPS test. The content is 0.085, 0.185, 0.525, 0.35 mol% respectively with the Ca content increasing. The emergence of aging phenomenon is explained through a defect dipole reorientation mechanism. Finally, the larger radius of Ca^2+^ ions is considered as a possible reason for the rapid polarization aging stability phenomenon. Our results may provide a possibility for accelerating aging treatment and thus make the utilization of the properties associated with the aging effect more efficient, stable and securer.

## Methods

### Preparation

The Ca, Ce hybrid doped BaTiO_3_ samples were prepared by separate doping Ca and Ce using a conventional solid state reaction method in this study. The separate doping samples Ba(Ti_0.92_Ce_0.08_)O_3_ (denoted as BTCe8) and (Ba_1-x_Ca_x_)TiO_3_ (x = 10, 20, 24, 30 mol%) (denoted as BCaxT) used the highly pure BaCO_3_ (99.9%), TiO_2_ (99.9%), as well as the analytically pure CeO_2_ (99.9%) and CaCO_3_ (99.9%) as starting materials. These highly pure and analytically pure chemicals are all from Alfa Aesar company. The hybrid doped BTCax + BTCe8 samples used the pre-sintered separate doping BTCe8 and BCaxT powders as starting materials.

Preparation method was as follows: (1) BTCe8 and BCaxT: the BaCO_3_, TiO_2_ and CeO_2_ (BTCe8) or the BaCO_3_, TiO_2_ and CaCO_3_ (BCaxT) were mixed and planet ball-milled in ethanol with agate ball mill media for 6 h and then dried. Then the mixture was calcined at 1300 °C for 4 h and then ball-milled for another 6 h and dried again; (2) BCaxT+BTCe8: through the step of (1), the calcined separate doping powders were gained, then these powder (for example BTCe8 and B20CaT) were used as the starting materials, then were planet ball-milled and mixed in proportion, and dried. The dried powder was added PVA aqueous solution (10 weight%) and then pressed into pellets with diameter of 12 mm. Sintering was done at 1400 °C with the holding time of 4 h in air.

### Characterization

The X-ray diffraction (XRD, X’Pert diffractometer (D8 Advance, Germany) with Cu Kα λ-1.5406 Å) was used to determine the phase structure of the ceramics at room temperature. Temperature dependence of the dielectric constant was measured from −150 to 200 °C at a frequency of 1k Hz. The time-dependent P*-*E hysteresis loops were characterized by Precision Premier II from Radiant Company, together with a high voltage amplifier. For the aging study, the samples were de-aged by heating to 200 °C for 2 h and then cooling to the room temperature to do the aging treatment. The aging time here was from several minutes to 1080 h. Hereafter the aging time of 1 min is denoted as the fresh sample state. Raman spectra were measured at room temperature using a HR800 Raman spectrometer (Horiba Jobin Yvon) with the 633 nm excitation. The X-ray photoelectron spectroscopy (XPS) spectra were obtained by Axis Ultra (UK) using monochromatic Al K_α_ (150W, 15 kV, 1486 eV).

## Additional Information

**How to cite this article**: Liu, S. *et al*. Rapid stability of ferroelectric polarization in the Ca, Ce hybrid doped BaTiO_3_ ceramics. *Sci. Rep.* **6**, 38354; doi: 10.1038/srep38354 (2016).

**Publisher's note:** Springer Nature remains neutral with regard to jurisdictional claims in published maps and institutional affiliations.

## Figures and Tables

**Table 1:** The stability time of the dielectric properties (under small signal) and the ferroelectric polarization (under large signal).

Methods	Ceramics	Stability time		Temperature	References
		Dielectric properties (under small signal)	Ferroelectric polarization (under large signal)		
Experiment	Mn doped BST	—	4 weeks, >100 h	Room, 25 °C	9, 14
	Mn doped BT	—	>238 h	60 °C	13
	Al doped BT	—	92 days	70 °C	30
	Fe, La doped BT	>100 min	—	25 °C	31
	Na, Sm doped BT	10^6^s, ~277 h	—	40 °C	32
	Hf, Zr doped BT	10^7^s, ~2777 h	—	20 °C	33
	Cu doped KNN	—	5 days	Room	23
	Fe doped PZT	—	>150 h, 277 h	125 °C, room	12, 34
	Fe doped PZT	>1000 min	—	80 °C	35
Simulation	Cu, Fe doped PT	—	Hours to days	Room	36

## References

[b1] Haertling G. H. Ferroelectric ceramics: history and technology. J. Am. Ceram. Soc. 82, 797–818 (1999).

[b2] Zhang S. J. & Li F. High performance ferroelectric relaxor-PbTiO_3_ single crystals: status and perspective. J. Appl. Phys. 111, 031301 (2012).

[b3] Bai Y., Han X., Zheng X. C. & Qiao L. Both high reliability and giant electrocaloric strength in BaTiO_3_ ceramics. Sci. Rep. 3, 2895 (2013). 24100662 10.1038/srep02895 PMC3792410

[b4] Liu X. & Tan X. Giant strains in non-textured (Bi_1/2_Na_1/2_)TiO_3_–based lead-free ceramics. Adv. Mater. 28, 574–578 (2016). 26596685 10.1002/adma.201503768

[b5] Genenko Y. A., Glaum J., Hoffmann M. J. & Albe K. Mechanisms of aging and fatigue in ferroelectrics. Mater. Sci. Eng. B 192, 52–82 (2015).

[b6] Zhou Y. . Modeling the paraelectric aging effect in the acceptor doped perovskite ferroelectrics: role of oxygen vacancy. J. Phys.: Condens. Matter 25, 435901 (2013). 24100273 10.1088/0953-8984/25/43/435901

[b7] Zhukov S. . Dynamics of polarization reversal in virgin and fatigued ferroelectric ceramics by inhomogeneous field mechanism. Phys. Rev. B 82, 014109 (2010).

[b8] Ren X. Large electric-field-induced strain in ferroelectric crystals by point-defect-mediated reversible domain switching. Nat. Mater. 3, 91–94 (2004). 14716304 10.1038/nmat1051

[b9] Zhang L. X., Chen W. & Ren X. Large recoverable electrostrain in Mn-doped (Ba,Sr)TiO_3_ ceramics. Appl. Phys. Lett. 85, 5658 (2004).

[b10] Liu W., Zhang L., Chen W., Li S. & Ren X. Large digital-characterized electrostrain in Mn-doped (Pb,Sr)TiO_3_ electro-shape-memory ceramics. Appl. Phys. Lett. 99, 092907 (2011).

[b11] Zhao X., Chen W., Zhang L. & Zhong L. The effect of the bipolar field on the aging behavior and the associated properties of the Mn-doped BaTiO_3_ ceramics. J. Alloys Comp. 618, 707–711 (2015).

[b12] Morozov M. I. & Damjanovic D. Hardening-softening transition in Fe-doped Pb(Zr,Ti)O_3_ ceramics and evolution of the third harmonic of the polarization response. J. Appl. Phys. 104, 034107 (2008).

[b13] Zhang L. . Mn dopant on the “domain stabilization” effect of aged BaTiO_3_ and PbTiO_3_-based piezoelectrics. Appl. Phys. Lett. 101, 242903 (2012).

[b14] Shi X., Wang J., Zhao Y., Liu S. & Zhang L. Competition effects of grain boundary and aging on the hysteresis loop behavior of (Ba_0.8_Sr_0.2_) (Ti,Mn)O_3_ ceramics. Ceram. Int. 42, 4734–4738 (2016).

[b15] Du G. . Internal bias field relaxation in poled Mn-doped Pb(Mn_1/3_Sb_2/3_)O_3_- Pb(Zr,Ti)O_3_ ceramics. Ceram. Int. 39, 7703–7708 (2013).

[b16] Zhang L. & Ren X. Aging behavior in single-domain Mn-doped BaTiO_3_ crystals: implication for a unified microscopic explanation of ferroelectric aging. Phys. Rev. B 73, 094121 (2006).

[b17] ZhangL., ErdemE., RenX. & EichelR. d. A. Reorientation of 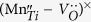 defect dipoles in acceptor-modified BaTiO3 single crystals: an electron paramagnetic resonance study. Appl. Phys. Lett. 93, 202901 (2008).

[b18] Xue D. . Aging effect in paraelectric state of ferroelectrics: implication for a microscopic explanation of ferroelectric deaging. Appl. Phys. Lett. 94, 082902 (2009).

[b19] Gao J. . Aging-induced two-step ferroelectric-to-paraelectric transition in acceptor- doped ferroelectrics. Appl. Phys. Lett. 96, 082906 (2010).

[b20] Gao J. H. . Aging-induced domain memory in acceptor-doped perovskite ferroelectrics associated with ferroelectric-ferroelectric transition cycle. Europhys. Lett. 96, 37001 (2011).

[b21] Yue Q., Luo L., Jiang X., Li W. & Zhou J. Aging effect of Mn-doped Ba_0.77_Ca_0.23_TiO_3_ ceramics. J. Alloys Comp. 610, 276–280 (2014).

[b22] Zhang Y. . The ageing and de-ageing behaviour of (Ba_0.85_Ca_0.15_) (Ti_0.9_Zr_0.1_)O_3_ lead-free piezoelectric ceramics. J. Appl. Phys. 118, 124108 (2015).

[b23] Lin D., Kwok K. W. & Chan H. L. W. Double hysteresis loop in Cu-doped K_0.5_Na_0.5_NbO_3_ lead-free piezoelectric ceramics. Appl. Phys. Lett. 90, 232903 (2007).

[b24] Yun S., Wang X., Shi J. & Xu D. Aging-induced double hysteresis loops in bismuth-doped (Ba,Ca)TiO_3_ ferroelectric ceramics. J. Mater. Res. 24, 3073–3077 (2011).

[b25] Puli V. S., Pradhan D. K., Riggs B. C., Chrisey D. B. & Katiyar R. S. Investigations on structure, ferroelectric, piezoelectric and energy storage properties of barium calcium titanate (BCT) ceramics. J. Alloys Comp. 584, 369–373 (2014).

[b26] Brajesh K., Kalyani A. K. & Ranjan R. Ferroelectric instabilities and enhanced piezoelectric response in Ce modified BaTiO_3_ lead-free ceramics. Appl. Phys. Lett. 106, 012907 (2015).

[b27] Lu D. Y., Han D. D., Sun X. Y., Zhuang X. L. & Zhang Y. F. Raman evidence for Ba-site Ce^3+^ in BaTiO_3_. Jpn. J. Appl. Phys. 52, 111501 (2013).

[b28] Wang H. & Wu J. Phase transition, microstructure, and electrical properties of Ca, Zr, and Sn-modified BaTiO_3_ lead-free ceramics. J. Alloys Comp. 615, 969–974 (2014).

[b29] Li C. X., Yang B., Zhang S. T., Zhang R. & Cao W. W. Effects of sintering temperature and poling conditions on the electrical properties of Ba_0.70_Ca_0.30_TiO_3_ diphasic piezoelectric ceramics. Ceram. Int. 39, 2967–2973 (2013).

[b30] Guo Y. Y., Qin M. H., Wei T., Wang K. F. & Liu J. M. Kinetics controlled aging effect of ferroelectricity in Al-doped and Ga-doped BaTiO_3_. Appl. Phys. Lett. 97, 112906 (2010).

[b31] Zhou C., Liu W. F. & Zhang L. X. Aging effect of point defects doped barium titanate. Key Eng. Mater. 519, 211–214 (2012).

[b32] Sareein T. . Dielectric aging behavior in A-site hybrid-doped BaTiO_3_ ceramics. Curr. Appl. Phys. 11, S90–S94 (2011).

[b33] Tura V. & Mitoseriu L. Ageing of low field dielectric constant and losses in (Hf, Zr)-doped BaTiO_3_ ceramics. Europhys. Lett. 50, 810–815 (2000).

[b34] Genenko Y. A. . Aging of poled ferroelectric ceramics due to relaxation of random depolarization fields by space-charge accumulation near grain boundaries. Phys. Rev. B 80, 224109 (2009).

[b35] Herbiet R., Tenbrock H. & Arlt G. The aging behaviour of the complex material parameters ε, d and s in ferroelectric PZT ceramics. Ferroelectrics 76, 319–326 (1987).

[b36] Erhart P., Träskelin P. & Albe K. Formation and switching of defect dipoles in acceptor-doped lead titanate: A kinetic model based on first-principles calculations. Phys. Rev. B 88, 024107 (2013).

[b37] Burns G. Lattice modes in ferroelectric perovskites. II. Pb_1-x_Ba_x_TiO_3_ including BaTiO_3_, Phys. Rev. B 10, 1951–1959 (1974).

[b38] Kchikech M. & Maglione M. Electronic and lattice excitations in BaTi0_3_: La. J. Phys.: Condens. Matter 6, 10159–10170 (1994).

[b39] Yao Z. . Structure and dielectric behavior of Nd-doped BaTiO_3_ perovskites. Mater. Chem. Phys. 109, 475–481 (2008).

[b40] Lu D. Y. . Self-compensation characteristics of Eu ions in BaTiO_3_. Solid State Ionics 201, 6–10 (2011).

[b41] Lu D. Y., Sun X. Y. & Toda M. A novel high-k ‘Y5V’ barium titanate ceramics co-doped with lanthanum and cerium. J. Phys. Chem. Solids 68, 650–664 (2007).

[b42] Paparazzo E. X-ray induced reduction effects at CeO_2_ surfaces: an x-ray photoelectron spectroscopy study. J. Vac. Sci. Technol., A 9, 1416–1420 (1991).

[b43] Koel B. E., Praline G., Lee H. I., White J. M. & Hance R. L. X-Ray photoelectron study of the reaction of oxygen with cerium. J. Electron Spectrosc. Relat. Phenom. 21, 31–46 (1980).

[b44] Lambeck P. V. & Jonker G. H. Ferroelectric domain stabilization in BaTiO_3_ by bulk ordering of defects. Ferroelectrics 22, 729–731 (1978).

[b45] Arlt G. & Neumann H. Internal bias field in ferroelectric ceramics: origin and time dependence. Ferroelectrics 87, 109–120 (1988).

[b46] Carl K. & Hardtl K. H. Electrical after-effects in Pb(Ti, Zr)O_3_ ceramics. Ferroelectrics 17, 473–486 (1978).

